# Microbiological and clinical characteristics of invasive Group B Streptococcal blood stream infections in children and adults from Qatar

**DOI:** 10.1186/s12879-022-07801-9

**Published:** 2022-11-24

**Authors:** Maisa Ali, Mohammed A. Alamin, Gawahir A. Ali, Khalid Alzubaidi, Bashir Ali, Abdellatif Ismail, Joanne Daghfal, Muna Almaslamani, Hamad Abdel Hadi

**Affiliations:** 1 Communicable Diseases Centre, Infectious Diseases Department, Hamad Medical Corporation, P. O. Box 3050, Doha, Qatar; 2 Internal Medicine Department, Hamad Medical Corporation, Doha, Qatar; 3 Paediatric Infectious Diseases, Sidra Medicine, Doha, Qatar

**Keywords:** Group B Streptococci (GBS), Strep agalactiae, Sepsis, Bacteraemia, Qatar

## Abstract

**Introduction:**

*Group B Streptococci* (GBS) colonize almost one third of human gastrointestinal and genitourinary tracts, particularly in females. The aim of this study is to evaluate the epidemiology, microbiological characteristics, and clinical outcomes of invasive GBS disease in Qatar from all age groups.

**Methods:**

A retrospective study was conducted on patients with confirmed GBS blood stream infections during the period between January 2015 and March 2019. Microbiological identification was performed using automated BD PhoenixTM system, while additional antimicrobial susceptibility tests were performed using E test and disc diffusion methods.

**Result:**

During the four years period, the incidence steadily rose from 1.48 to 2.09 cases per 100.000 population. Out of 196 confirmed cases of invasive GBS infections, the majority were females (63.7%, 125/196) of which 44.8% were pregnant and 53.6% were colonized. Three distinct affected age groups were identified: children ≤ 4 years of age (35.7%), young adults 25–34 (20.9%) and the elderly ≥ 65 year (17.4%). Presenting symptoms were mild with fever in 53% of cases while 89% of cases had Pitt bacteraemia score of ≤ 2. Isolates were universally sensitive to penicillin, ceftriaxone, and vancomycin at 100% but with significant resistance to erythromycin (49%) and clindamycin (28.6%) while 16.8% had inducible clindamycin resistance. Clinical outcomes showed cure rate of 87.25% with complications in (8.76%) and 4% mortality.

**Conclusion:**

There is a rising trend of Group B Streptococcal blood stream infections in Qatar with significantly high clindamycin and erythromycin resistance rates. Universal susceptibility rates were demonstrated for penicillin, ceftriaxone, and vancomycin.

**Supplementary Information:**

The online version contains supplementary material available at 10.1186/s12879-022-07801-9.

## Introduction

*Group B Streptococci* (GBS) are gram-positive cocci that commonly colonize the gastrointestinal and genitourinary tracts of adults particularly of females and pregnant women [1]. Despite being harmless in the majority of colonized individuals, the pathogen is capable of causing invasive diseases primarily in neonates, infants, pregnant and postpartum women as well as the elderly with significant morbidity and mortality [2].

The spectrum of the invasive disease including maternal and neonatal sepsis, was recognised during the 1960s which led to major public health measures to improve recognition, management and prevention of GBS infections [3]. For pregnant women and neonates, risks of perinatal transmission are highest in the late pregnancy and perinatal periods mainly during vaginal delivery, delayed rupture of amniotic membranes and preterm labour. Based on unfavourable outcomes as well as cost effectiveness, the recognised public health challenge led to the non-uniform clinical practise of screening pregnant women at late pregnancies to timely identify carriers [2, 4, 5]. To avert potential subsequent morbidity and mortality, in the USA, the Centers for Disease Control and Prevention (CDC) recommend universal screening of pregnant women in late trimester (between week 35 and 37 of gestation) then apply preventive measures including intrapartum antimicrobial prophylaxis while the European guidelines follow a similar approach or more frequently identify high risk cases based upon prior risk assessment [6].

The global epidemiology of GBS disease is variable since the average estimate of maternal colonization is about 20% with regional variations ranging between 11% (lower estimates) and 35% (higher estimates), with lower prevalence in Southern and Eastern Asia at 11% and 12.5% respectively [7].

Similarly, the pattern of the distribution of the GBS disease is also evolving since younger adults including non-pregnant women and the elderly are recognized vulnerable groups. Epidemiological studies revealed no overall gender or ethnic preponderance albeit some studies showed inclination towards males and black ethnicity. Furthermore, research studies have identified multiple host and pathogen contributing risk factors such as chronic comorbidities, obesity and immunosuppression in addition to prevalence of certain serotypes of GBS associated with invasive virulent factors [8, 9]. Conversely, antimicrobial susceptibilities of GBS are also showing regional variations with clindamycin and erythromycin resistance estimated around 25%, being highest in Asia when compared to Western reports [7, 9]. Although studies from different parts of the world have reported universal susceptibility of GBS to B-lactam antibiotics including penicillin, antimicrobial susceptibility testing for alternative antimicrobial options remains crucial in case of allergies, adverse events, or intolerance. For example, penicillin allergy were reported in 10% of pregnant woman which is certainly higher if were to include drug intolerance or adverse events. [10]. Therefore, advising for alternative presumptive management, entails evaluation of the local antimicrobial characteristics and resistance patterns. As a comparison, previous studies of invasive GBS disease from the United States, demonstrated the prevalence of antimicrobial resistance ranging from 25 to 32% for erythromycin and from 13 to 20% for clindamycin [11, 12]. Regionally a study of invasive maternal GBS disease, highlighted a noticeable rising antimicrobial resistance pattern including erythromycin and clindamycin at 12.6 and 7% respectively [13]. Despite these observations, the scale of the problem might be underestimated since appropriate methodologies for susceptibility testing were not fully evaluated. For example, inducible clindamycin resistance might develop in isolates that were apparently phenotypically susceptible [14, 15].

In order to investigate the extent of the problem in Qatar, we evaluated the antimicrobial susceptibility profiles of GBS.

### Settings, material, methods and statistical analysis

Hamad Medical Corporation (HMC) is the main healthcare provider at the state of Qatar serving a population of around 2.7 million through acute and specialized hospitals with a total bed capacity of almost 2500 beds and 8 intensive care units, encompassing four acute care hospitals and six specialised hospitals (Additional file 1: Appendix 1). The retrospective study was conducted on patients registered at HMC between January 2015 and March 2019. All consecutive microbiologically confirmed GBS blood stream infection cases were recorded, identified then analysed. Electronic Hospital Information System (HIS) was used to collect patients’ demographics, microbiological as well as clinical characteristics including outcomes.

### Case definition, microbiological identifications, and antimicrobial susceptibility testing

We defined invasive blood stream infection (BSI) as at least one positive blood culture for *group B streptococcus* from evaluated patients exhibiting probable signs of infection. In patients with persistent BSIs caused by the same organism, only the first episode was included. If patients had more than one episode of BSIs during the same admission period, only the first infection episode is counted for prevalence analysis but repeated ones are included for microbiological characteristics and clinical outcomes.

Microbial identifications and antimicrobial susceptibility tests were performed using standard methods that included automated bacterial isolation using BD Phoenix system while identification and confirmation were performed using Matrix-Assisted Laser Desorption Ionization Time of Flight Mass Spectrometry (MALDI-TOF MS) of Bruker Daltonics MALDI Biotyper (Billerica, MA, USA) according to the manufacturer’s recommendations. E test and disc diffusion methods were used for antibiotic susceptibility tests (AST) according to the Clinical Laboratory Standard Institute (CLSI) guidelines while D test was additionally performed and interpreted to detect inducible resistance for erythromycin—clindamycin discordant isolates. The hospitals central laboratory complies with modern and updated standards including international inspection and accreditation [16].

### Statistical analysis

Statistical analysis was performed using Stata statistical software, version 16.1 (Stata Corporation, College Station, TX, USA). Continuous variables were presented as mean and range while categorical variables were presented as total number and percentages. Calculation of the annual incidence was based upon total yearly identified numbers divided by the country population at that time.

## Results

**Fig. 1:**
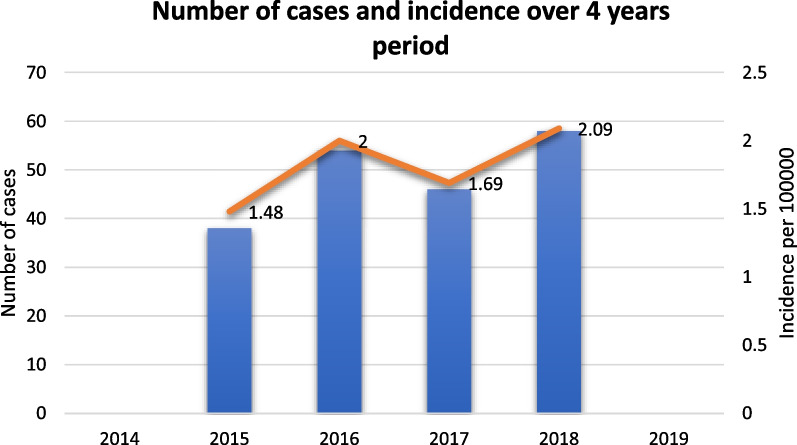
Incidence trends of GBS bacteraemia in the state of Qatar from 2015 to 2018

Of the 200 GBS identified patients, 4 were excluded for not fulfilling inclusion criteria (positive CSF with negative blood cultures). Over the four years study period, analysis was performed on 196 unique episodes of GBS blood stream infections showing rising trends of the invasive disease from incidence of 1.48 per 100.000 population in the first year to 2.09 per 100.000 in the last year (Fig. 1). The majority of cases were females (F 63.8%, 125/196 and M 36.2%, 71/196) from three distinct age groups: paediatric ≤ 4 years at 35.7% (70/196) (predominately neonates, n 39, 59% infected ≤ 7 days), followed by young adults between 25 and 34 year of age at 20.9% (41/196) and elderly patients (> 65 year) at 17.4% (34/196) while others were 26% (51/196) (Table 1). In addition to the BSI, 72 isolates of GBS were concomitantly isolated from other single or multiple sites of which 68% (49/72) were classified as isolated from non-sterile sites such as the urinary tract (n = 17), placenta cultures (n = 10), both urine and placenta (n = 13), wounds (n = 7) and sputum samples (n = 2) while isolates from sterile sites include CSF in 28% (20/72) and synovial fluid at 4.2% (3/72).

**Table 1:** Demographic data for patients with invasive Group B streptococcal infection

	Total (N = 196)
Gender	
Female	125 (63.78%)
Male	71 (36.22%)
Pregnancy	56/125 (45%)
Pregnant ≤ 35 years	41/56 (73%)
Pregnant > 35 years	15/56(27%)
Age	30.42 (27.44)
0–4 years	70 (35.71%)
5–14 years	2 (1.02%)
15–24 years	8 (4.08%)
25–34 years	41 (20.92%)
35–44 years	18 (9.18%)
45–54 years	9 (4.59%)
55–64 years	14 (7.14%)
65 + years	34 (17.35%)
Nationality	
Expatriates	135 (68.88%)
Qatari nationals	61 (31.12%)
Comorbidities	
Diabetes mellitus	19 (9.69%)
Gestational diabetes	9 (4.59%)
Chronic kidney diseases	1 (0.51%)
Chronic liver diseases	2 (1.02%)
Connective tissue diseases	14 (7.14%)
Combination of risk factors	45 (22.96%)
No pre-existing medical condition	106 (54.08%)
Pitt Bacteraemia Score	
Low (0–2)	176(89.79%)
Moderate (3–5)	11 (5.61%)
Sever (> / 6)	9 (4.59%)
Symptoms of GBS infection	
Fever	104 (53.06%)
Skin and Soft tissues infections	12 (6.12%)
CNS symptoms	5 (2.55%)
Respiratory symptoms	5 (2.55%)
Any combination	65 (33.16%)
Urinary symptoms	1 (0.51%)
No symptoms	4 (2.04%)
Sources-Sterile sites include:	
CSF	20 (10.20%)
Synovial fluid	3 (1.53%)
Sources-Nonsterile sites include:	
Placenta	10 (5.10%)
Urine	17 (8.67%)
Urine and placenta	13 (6.63%)
Wound	7 (3.57%)
Sputum	2 (1.02%)

*Data are presented as numbers and percentages for categorical variables and as mean and SD for continuous variables

*CSF* cerebrospinal fluid, *CNS* central nervous system

Review of clinical symptoms associated with GBS BSI, fever was the most common presenting symptoms albeit modest for the invasive disease at 53% (104/196), while 89.8% of patients (176/196) had Pitt bacteraemia score of ≤ 2. More than half of patients have no underlying medical conditions (54%, 106 /196) while diabetes mellitus was one of the most common comorbid conditions at 9.69% (19/196) (Table 1).

Regarding prevalence and previous documented colonization in female patients. Out of the 125 female cases, 45% were pregnant (56/125) of which evidence of GBS colonization was detected in more than half of them (53.6%, 30/56), however timing of the bacteraemia preceded antimicrobial prophylaxis.

For pregnant women, history of previous penicillin allergy was documented in only 3.6% of cases (7/56) but only two had documentation of previous anaphylaxis. Penicillin and cephalosporin were the most common antibiotics used for treatment while alternative therapy was only used in 9% of cases (clindamycin and vancomycin). Assessment of the outcome of management of the invasive disease showed high cure rates of 87.25%, secondary complications of 8.76% and overall, 30-day mortality of 4% while distinct neonatal outcome of the 56 infected pregnant women showed complication in 32% of cases.

### Antimicrobial susceptibility testing

**Fig. 2:**
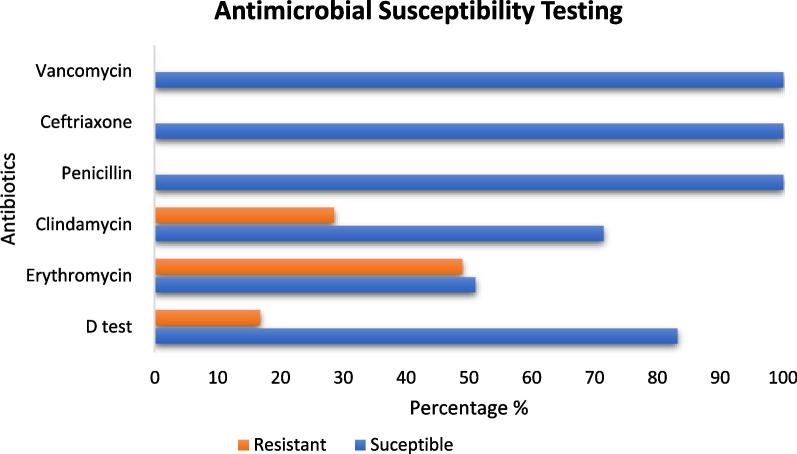
Antibiotic Susceptibility Testing for invasive GBS bacteraemia isolates

To assess microbiological characterises and ASTs, E test (for penicillin, ceftriaxone and vancomycin), and disc diffusion methods (for clindamycin and erythromycin) were performed showing universal penicillin, ceftriaxone and vancomycin sensitivity at 100% (196/196) while clindamycin susceptibility was recorded at 71% (140/196) and erythromycin susceptibility at 51% of isolates (100/196). There were 33 isolates (overall 16.8% and 23.6% of clindamycin susceptible isolates) that showed inducible clindamycin resistance through D test (Fig. 2). Details of microbiological details are provided as Additional file 1: Appendix 2.

## Discussion

Despite being a commensal organism in almost one third of the adult population, GBS is capable of causing invasive diseases such as sepsis, urinary, respiratory, central nervous system, bone and soft tissues as well as blood stream infections (BSI) with significant morbidity and mortality [17, 18].

Our four-year study showed rising trends of the BSI invasive disease from 1.4 per 100.000 persons at first year culminating to 2.09 per 100.000 population in the last year, nevertheless it remains almost half prevalent when compared to Western public health surveillance [18–20]. This highlighted observation might support postulations that the low incidence of GBS invasive disease in Asian countries might be secondary to distinct clonal distribution, host genetic factors or pathogen characteristics which favour colonisation with less invasive serotypes [21, 22].

Like other related studies, our results showed clear female preponderance almost half of them were pregnant and concomitantly colonised [17, 23]. This augments previous established knowledge of higher risks of invasive GBS disease during pregnancy and perinatal period because of multiple host vulnerabilities and pathogen characteristics [17, 24]. Similarly, the study also affirms that there are three distinct affected populations with BSI, namely neonates and young children, young adults including pregnant women, and the elderly. There are plausible explanations since previous observational studies linked invasive GBS disease with prematurity, prolonged rupture of amniotic membranes, maternal colonization, high bacterial load, sexually transmitted infections, immune dysfunction including neoplastic diseases as well as old age [25–27]. These observations are also in line with global research which demonstrated similar prevalence of the highlight age groups when compared to others [18, 19]. 55.7%of the affected children were neonates (< 28 days) of whom 59% affected by early onset disease ≤ 7 days of age. Comparably, older age groups (17.4% in ≥ 65 year) are a sizeable cohort of the studied population which is probably linked to multiple comorbidities, waning of the immunity, immunosenescence as well as association with the expected age related neoplastic diseases [9, 28, 29]. Of note, in nearly 38% of cases of invasive GBS, the bacteria were isolated from multiple sites such as the placenta, urine and wounds which postulates that the invasive disease is subsequently followed by ascending progressive or metastatic infections.

It is reassuring that while examining microbiological characteristics it demonstrates universal susceptibility to penicillin, ceftriaxone and vancomycin at 100%, since the historic antibiotic, penicillin remains the first choice for presumptive management with no globally observed resistance [30]. In clinical practise, it is axiomatic that penicillin resistance for *Group B streptococci* is extremely rare, attributed to a stable Penicillin Binding Protein (PBP) targets when compared to mutated ones for other resistant gram-positive bacteria [31]. This is supported by the extremely rare isolation of extensively resistant strains from Japan harbouring mutant PBPs leading to penicillin and cephalosporin antimicrobial resistance [32, 33]. In contrast, alternative antimicrobials that act at alternative cellular sites such as macrolides and lincomycin represented by erythromycin and clindamycin showed significant resistance at 49% and 29% respectively, which is significantly higher than other related regional or global studies [7, 12, 13, 34]. Furthermore, performed D test through disc diffusion methods, demonstrated that almost a quarter of phenotypically clindamycin susceptible isolates possess inducible resistance adding to the recommendations that apparent clindamycin susceptibilities should be taken with caution because of potential underlying genotypic resistance [14, 35]. In patients with penicillin allergy, local and international recommendation is to treat potential invasive disease presumptively with lincomycin or glycopeptides such as clindamycin or vancomycin sometimes without confirmatory susceptibilities [2, 5, 36]. The Centers for Disease Control and Prevention (CDC) in the USA recommend universal screening of pregnant women in late trimester while the revised guidelines of 2010 states that penicillin-allergic women at high risk for anaphylaxis should receive clindamycin provided their GBS isolates are susceptible to both clindamycin and erythromycin determined by a reliable antimicrobial susceptibility test methods. Importantly, if isolates are sensitive to clindamycin but resistant to erythromycin, the guidelines states that it can only be used if clindamycin inducible resistance is negative while erythromycin is no longer recommended as an alternative therapy. Furthermore, penicillin-allergic women at high risk for anaphylaxis should receive vancomycin as presumptive therapy when susceptibilities are unknown or if their isolates are intrinsically resistant to clindamycin or demonstrate inducible resistance determined through reliable antimicrobial susceptibility tests [6].

To avoid confusing medical practitioners, taking into consideration the high examined rates of in-vitro resistance to clindamycin as well as considerable inducible resistance, we recommend reviewing guidelines for empirical therapy to avoid clindamycin when possible and only use it following establishing susceptibilities. Based on argued evidence, recommended alternatives can be limited to 3rd generation cephalosporins or glycopeptides (Fig. 2).

Regarding clinical presentation and outcomes, it is intriguing that the invasive infection has mild presentation with fever only reported in about half of the cases in all the age groups. This must be examined with caution since at the extremes of age, infection can present in the absence febrile symptoms [37, 38]. Likewise, to assess for complication of invasive BSI, almost 90% of cases had low Pitt bacteraemia score (≤ 2) reflected on high cure rates approaching 90% with relatively low rate of complications (8.8%) as well as low 30-day mortality (4%) encompassing all age groups. Nevertheless, although the observed mortality of invasive GBS is low, it continues to raise the alarms for infection and public health specialists, since it affects neonates and young adults. Of note, in our study although the invasive disease has not been linked to significant clinical outcomes in pregnant women, it has been associated with substantial neonatal complications in 38% of cases. That probably stems from the firm association between GBS colonization, invasive disease and preterm labour which is associated higher rates of neonatal complications [39]. This should add to the argument of the importance of the control and prevention measures for GBS disease including timing of screening, use of more sensitive molecular methods, type and duration of antimicrobial prophylaxis as well as the urgent need for the development of future targeted vaccination programs which are at advanced stages of development. [4, 40–42].

## Conclusion

Our study from Qatar of nearly 200 cases of invasive GBS blood stream infection from all age groups over four years period, demonstrated lower but rising incidence rates compared to other parts of the world, females’ preponderance with three distinct populations: neonates, young adults including pregnant women and the elderly. Presenting symptoms were mild with relatively low complications and 30-day mortality. GBS remains highly susceptible to penicillin but with significant clindamycin resistance which should be reviewed as empirical or tailored therapy.

## Supplementary Information

**Additional file 1. Appendix 1: **Hospitals Covered by Hamad Medical Corporation (HMC) in Qatar. **Appendix 2:** Antibiotic Susceptibility and D Tests for 196 invasive GBS bacteraemia isolates.

## Data Availability

Strict measures were observed for data collection as well as management during the study. Data were not shared at any level with any individuals that are not connected to the research project and mainly handled by the Primary Investigators. Anonymised data can be made available upon request to the authors following permission from the Medical Research Centre at HMC. The MRC at HMC abides by the rules and regulations of data safety measures in scientific research and prohibits any unauthorised use of confidential data.

## References

[1] Furfaro LL, Chang BJ, Payne MS. Perinatal Streptococcus agalactiae epidemiology and surveillance targets. Clin Microbiol Rev. 2018;31(4). 10.1128/CMR.00049-18 PMC6148196 30111577

[2] Raabe VN, Shane AL (2019). Group B Streptococcus (Streptococcus agalactiae). Microbiol Spectr.

[3] Mannik M, Baringer JR, Stokes J (1962). Infections due to group B beta-hemolytic streptococci: report of three cases and review of the literature. N Engl J Med.

[4] Is Prenatal Screening for GBS Cost-Effective in the United States? OB/GYN clinical alert. 2021;38(8).

[5] Di Renzo GC, Melin P, Berardi A, Blennow M, Carbonell-Estrany X, Donzelli GP (2015). Intrapartum GBS screening and antibiotic prophylaxis: a European consensus conference. J Matern Fetal Neonatal Med.

[6] Verani JR, McGee L, Schrag SJ (2010). Prevention of perinatal group B streptococcal disease–revised guidelines from CDC, 2010. MMWR Recomm Rep.

[7] Huang J, Li S, Li L, Wang X, Yao Z, Ye X (2016). Alarming regional differences in prevalence and antimicrobial susceptibility of group B streptococci in pregnant women: a systematic review and meta-analysis. J Glob Antimicrob Resist.

[8] Navarro-Torné A, Curcio D, Moïsi JC, Jodar L (2021). Burden of invasive group B Streptococcus disease in non-pregnant adults: a systematic review and meta-analysis. PLoS ONE.

[9] Francois Watkins LK, McGee L, Schrag SJ, Beall B, Jain JH, Pondo T (2019). Epidemiology of invasive group B Streptococcal infections among nonpregnant adults in the United States, 2008–2016. JAMA Intern Med.

[10] Blumenthal KG, Shenoy ES (2020). Penicillin allergy in pregnancy. JAMA.

[11] Borchardt SM, DeBusscher JH, Tallman PA, Manning SD, Marrs CF, Kurzynski TA (2006). Frequency of antimicrobial resistance among invasive and colonizing Group B streptococcal isolates. BMC Infect Dis.

[12] Castor ML, Whitney CG, Como-Sabetti K, Facklam RR, Ferrieri P, Bartkus JM (2008). Antibiotic resistance patterns in invasive group B Streptococcal Isolates. Infect Dis Obstet Gynecol.

[13] Boswihi SS, Udo EE, Al-Sweih N (2012). Serotypes and antibiotic resistance in Group B streptococcus isolated from patients at the Maternity Hospital, Kuwait. J Med Microbiol.

[14] Tang P, Ng P, Lum M, Skulnick M, Small GW, Low DE (2004). Use of the vitek-1 and vitek-2 systems for detection of constitutive and inducible macrolide resistance in group B Streptococci. J Clin Microbiol.

[15] Richter SS, Howard WJ, Weinstein MP, Bruckner DA, Hindler JF, Saubolle M (2007). Multicenter evaluation of the BD phoenix automated microbiology system for antimicrobial susceptibility testing of Streptococcus species. J Clin Microbiol.

[16] Humphries RM, Ambler J, Mitchell SL, Castanheira M, Dingle T, Hindler JA, et al. CLSI methods development and standardization working group best practices for evaluation of antimicrobial susceptibility tests. J Clin Microbiol. 2018;56(4). 10.1128/JCM.01934-17 PMC5869819 29367292

[17] Raabe VN, Shane AL. Group B Streptococcus (Streptococcus agalactiae). Microbiol Spectr. 2019;7(2). 10.1128/microbiolspec.gpp3-0007-2018 PMC6432937 30900541

[18] Ballard MS, Schønheyder HC, Knudsen JD, Lyytikäinen O, Dryden M, Kennedy KJ (2016). The changing epidemiology of group B streptococcus bloodstream infection: a multi-national population-based assessment. Infect Dis (Lond).

[19] Collin SM, Shetty N, Lamagni T (2020). Invasive group B Streptococcus infections in adults, England, 2015–2016. Emerg Infect Dis.

[20] Laupland KB, Pasquill K, Parfitt EC, Steele L (2019). Bloodstream infection due to β-hemolytic streptococci: a population-based comparative analysis. Infection.

[21] Russell NJ, Seale AC, O'Driscoll M, O'Sullivan C, Bianchi-Jassir F, Gonzalez-Guarin J (2017). Maternal colonization with group B Streptococcus and serotype distribution worldwide: systematic review and meta-analyses. Clin Infect Dis.

[22] Shabayek S, Spellerberg B (2018). Group B Streptococcal colonization, characteristics, and epidemiology molecular. Front Microbiol.

[23] Le Doare K, Heath PT (2013). An overview of global GBS epidemiology. Vaccine.

[24] van Kassel MN, Janssen S, Kofman S, Brouwer MC, van de Beek D, Bijlsma MW (2021). Prevalence of group B streptococcal colonization in the healthy non-pregnant population: a systematic review and meta-analysis. Clin Microbiol Infect.

[25] Vuillemin X, Hays C, Plainvert C, Dmytruk N, Louis M, Touak G (2021). Invasive group B Streptococcus infections in non-pregnant adults: a retrospective study, France, 2007–2019. Clin Microbiol Infect.

[26] Berardi A, Trevisani V, Di Caprio A, Bua J, China M, Perrone B (2021). Understanding factors in group B Streptococcus late-onset disease. Infect Drug Resist.

[27] Shelburne SA, Tarrand J, Rolston KV (2013). Review of streptococcal bloodstream infections at a comprehensive cancer care center, 2000–2011. J Infect.

[28] High KP, Edwards MS, Baker CJ (2005). Group B Streptococcal infections in elderly adults. Clin Infect Dis.

[29] Ho CM, Chi CY, Ho MW, Chen CM, Liao WC, Liu YM (2006). Clinical characteristics of group B streptococcus bacteremia in non-pregnant adults. J Microbiol Immunol Infect.

[30] Hayes K, O'Halloran F, Cotter L (2020). A review of antibiotic resistance in Group B Streptococcus: the story so far. Crit Rev Microbiol.

[31] van der Linden M, Mamede R, Levina N, Helwig P, Vila-Cerqueira P, Carriço JA (2020). Heterogeneity of penicillin-non-susceptible group B streptococci isolated from a single patient in Germany. J Antimicrob Chemother.

[32] Seki T, Kimura K, Reid ME, Miyazaki A, Banno H, Jin W (2015). High isolation rate of MDR group B streptococci with reduced penicillin susceptibility in Japan. J Antimicrob Chemother.

[33] Kitamura M, Kimura K, Ido A, Seki T, Banno H, Jin W (2019). Relatively high rates of cefotaxime- and ceftriaxone-non-susceptible isolates among group B streptococci with reduced penicillin susceptibility (PRGBS) in Japan. J Antimicrob Chemother.

[34] Murdoch DR, Reller LB (2001). Antimicrobial susceptibilities of group B streptococci isolated from patients with invasive disease: 10-year perspective. Antimicrob Agents Chemother.

[35] Desjardins M, Delgaty KL, Ramotar K, Seetaram C, Toye B (2004). Prevalence and mechanisms of erythromycin resistance in group A and group B Streptococcus: implications for reporting susceptibility results. J Clin Microbiol.

[36] Paccione KA, Wiesenfeld HC (2013). Guideline adherence for intrapartum group B streptococci prophylaxis in penicillin-allergic patients. Infect Dis Obstet Gynecol.

[37] Wang ME, Neuman MI, Nigrovic LE, Pruitt CM, Desai S, DePorre AG (2021). Characteristics of afebrile infants ≤60 days of age with invasive bacterial infections. Hosp Pediatr.

[38] High KP, Bradley SF, Gravenstein S, Mehr DR, Quagliarello VJ, Richards C (2009). Clinical practice guideline for the evaluation of fever and infection in older adult residents of long-term care facilities: 2008 update by the Infectious Diseases Society of America. Clin Infect Dis.

[39] Bianchi-Jassir F, Seale AC, Kohli-Lynch M, Lawn JE, Baker CJ, Bartlett L (2017). Preterm birth associated with group B Streptococcus maternal colonization worldwide: systematic review and meta-analyses. Clin Infect Dis.

[40] Kobayashi M, Schrag SJ, Alderson MR, Madhi SA, Baker CJ, Sobanjo-Ter Meulen A (2019). WHO consultation on group B Streptococcus vaccine development: report from a meeting held on 27–28 April 2016. Vaccine.

[41] Hahn BA, de Gier B, van Kassel MN, Bijlsma MW, van Leeuwen E, Wouters MGAJ (2021). Cost-effectiveness of maternal immunization against neonatal invasive Group B Streptococcus in the Netherlands. Vaccine.

[42] Carreras-Abad C, Ramkhelawon L, Heath PT, Le Doare K (2020). A vaccine against group B Streptococcus: recent advances. Infect Drug Resist.

