# Supplementary material for: Microbiological and clinical characteristics of invasive Group B Streptococcal blood stream infections in children and adults from Qatar
Source: BMC Infect Dis. 2022 Nov 24;22:881. doi: 10.1186/s12879-022-07801-9 (PMC9701022; doi:10.1186/s12879-022-07801-9)
Supplement: Supplementary file 1 — Additional file 1. Appendix 1: Hospitals Covered by Hamad Medical Corporation (HMC) in Qatar. Appendix 2: Antibiotic Susceptibility and D Tests for 196 invasive GBS bacteraemia isolates. [file 12879_2022_7801_MOESM1_ESM.docx]

**Appendix 1: Hospitals Covered by Hamad Medical Corporation (HMC) in Qatar**

| **Hospital** | **Bed Capacity** | **Specialty** |
| --- | --- | --- |
| Hamad General Hospital (HGH) | 605 | Acute care general hospital with three specialized intensive care units |
| Rumaila Hospital (RH) : | 620 | Acute geriatrics and long-term facility hospital |
| The Surgical Specialty Centre (SSC) | 320 | Acute and specialized surgical care center |
| The Heart Hospital (HH) | 120 | Specialized cardiac and cardiothoracic hospital |
| The Women Wellness and Research Centre (WWRC) | 220 | Specialized women wellness hospital |
| Communicable Diseases center (CDC) | 65 | Dedicated communicable diseases hospital with full capacity isolation and negative pressure rooms |
| The National Centre for Cancer and Research (NCCR) | 65 | Specialized hospital for solid organs and hematological malignancies including bone marrow transplantation |
| Ambulatory Care Center (ACC) : | Ambulatory care | Specialized short stay hospital that houses, day care gastroenterology, urology, ophthalmology and ENT services |
| Hazem Mubairek General Hospital (HMGH) | 120 | Acute care hospital at the south west of the city |
| Wakra Hospital (WH) | 320 | Acute care at the south of the city |

**Appendix 2: Antibiotic Susceptibility and D Tests for 196 invasive GBS bacteraemia isolates**

| Antimicrobials N and % | Penicillin | Ceftriaxone | Vancomycin | Erythromycin | Clindamycin |
| --- | --- | --- | --- | --- | --- |
|  |  |  |  |  |  |
| Susceptible | 196(100%) | 196(100 %) | 196 (100 %) | 100 (51 %) | 140(71.4%) |
| Resistant | 0 | 0 | 0 | 96 (49 %) | 56(28.6%) |
| Total Isolates | 196 | 196 | 196 | 196 | 196 |
| *Positive D test | 33 Isolates |  |  |  | Overall, 16.8 % (33/196) |
|  |  |  |  |  | 23.6 % of phenotypic Clindamycin susceptible isolates (33/140) |

*For erythromycin -clindamycin discordant isolates to detect clindamycin inducible resistance
